# Putting Yourself in the Skin of In- or Out-Group Members: No Effect of Implicit Biases on Egocentric Mental Transformation

**DOI:** 10.3389/fpsyg.2019.01338

**Published:** 2019-06-14

**Authors:** Gianluca Saetta, Peter Brugger, Hannah Schrohe, Bigna Lenggenhager

**Affiliations:** ^1^Neuropsychology, Department of Neurology, University Hospital Zurich, Zurich, Switzerland; ^2^Cognitive Neuropsychology, Department of Psychology, University of Zurich, Zurich, Switzerland; ^3^Neuropsychology Unit, Valens Rehabilitation Centre, Valens, Switzerland

**Keywords:** egocentric mental transformation, implicit biases, embodied cognition, perspective taking, motor imagery

## Abstract

Previous studies suggest that visual encoding of ethnicity of in-group/out-group members might influence empathy and sensorimotor sharing. Here, we investigated whether mental perspective taking, presumably a precursor of empathy, is also influenced by in-group/out-group perception and the implicit attitudes toward it. We used an embodied egocentric visual-perspective taking task, the full body rotation task (FBR), in which participants were asked to mentally rotate themselves into the position of dark- or light-skinned bodies. FBR was contrasted to a pure sensorimotor task, the hand laterality task (HLT), in which participants were asked to mentally rotate their hand to the posture of seen light- or dark-skinned hands, which does not require mental simulation of another person’s perspective. We expected the FBR but not the HLT to be influenced by the skin color of the stimuli and by the individual implicit biases toward out-group members. Contrary to this hypothesis, we found that neither skin color nor implicit biases modulated reaction times (RTs) in either task. The data thus suggest that unlike other empathy tasks, skin color does not influence visuospatial perspective taking.

## Introduction

To understand other people’s actions, intentions, and emotions, we presumably unconsciously simulate their perspective and map their bodily states through our own sensorimotor system (termed “sensorimotor resonance”; but see also Vannuscorps et al., 2013). These simulation processes are considered an important basis for social cognition and prosocial behavior (Bernhardt and Singer, 2012) and are modulated by interpersonal perception. Stronger sensorimotor resonance is found when observing in-group as compared to out-group members (Azevedo et al., 2012). From an evolutionary perspective, such differential behavior, which is often mediated by visual features (e.g., skin color), might have been important for individuals to identify coalitional alliances (Kurzban et al., 2001). The magnitude of group-dependent simulation has further shown to correlate with the observer’s implicit biases toward that specific in- or out-group members (Avenanti et al., 2010).

Various measures of sensorimotor simulation have been explored, such as corticospinal excitability during pain observation (Avenanti et al., 2010), neural activation in the cortical pain matrix (Cunningham et al., 2004; Berlingeri et al., 2016), sensory remapping of touch (Fini et al., 2013), or joint action (Sacheli et al., 2015). During these, the out-group versus in-group person’s or avatar’s face (Fini et al., 2013), body parts (e.g., hands – Avenanti et al., 2010), or full body (Sacheli et al., 2015) was observed from a third-person perspective. Participants thus presumably took the other person’s perspective (by performing a mental transformation task) to infer and simulate their motor state. Surprisingly however, to our knowledge, no study has directly assessed the interaction between the other person’s skin color and a spatial egocentric perspective taking task. Here, we investigated how the skin color of a seen body and implicit biases toward in-group/out-group members, as measured by the implicit association task (Greenwald et al., 1998), influence RT in a FBR. Participants made laterality judgments for light- or dark-skinned avatars viewed from different angles, by putting themselves in the spatial perspective of another person and deciding from this perspective whether the left or right hand was marked. This task has shown to recruit areas in the TPJ (e.g., Zacks et al., 2003; Ganesh et al., 2015; van Elk et al., 2017), a crucial hub in the empathy-related network (review in Singer, 2006). Accordingly, perspective taking tasks are modulated by emotional state and trait empathy (Thakkar et al., 2009; Mohr et al., 2010). Furthermore, a mutual link between such spatial perspective taking and interpersonal perception has been suggested (Erle and Topolinski, 2017; van Elk et al., 2017). We thus predicted a facilitation of mental body transformations (i.e., faster RTs) toward the perspective of an in-group (i.e., light-skinned) as compared to an out-group (dark-skinned) member. We expected this effect to be positively correlated with implicit biases.

We contrasted the behavior in the FBR to an egocentric sensorimotor simulation task, i.e., the HLT (Parsons, 1987), which requires motor simulation, but no mental perspective taking of another person. Here, participants mentally rotate their own hand from an egocentric perspective into the postures of the seen hand and decide whether a left or a right hand, displayed at different angles of rotation, is presented. A wealth of neuroimaging studies suggest that this task recruits distinct neural networks with respect to the FBR, as they pinpointed performance on the HLT onto sensorimotor network regions, including frontal (the lateral premotor cortex and the supplementary motor area) and parietal (superior parietal lobule and intraparietal sulcus) areas (Bonda et al., 1995; Parsons et al., 1995; Vingerhoets et al., 2002; Wraga et al., 2003; Zapparoli et al., 2016; Gandola et al., 2017). Behaviorally, the time needed to provide these cognitive perceptual decisions seems to be affected by biomechanical constraints or handedness (Mellet et al., 2016). For these reasons, we predicted no influence of skin color and/or implicit bias toward out-groups on the HLT.

## Materials and Methods

### Participants

Only light-skinned and Caucasian participants, reporting no African nor Asian ancestries, participated in the study after signing the online informed consent form. They were 47 right-handed individuals (male: 24, female: 23; mean age: 30.83 ± 10.26; Italian speakers: 24; German speakers: 23).

### Experimental Procedure

An online survey built with “Psytoolkit”^1^ included demographic questions (e.g., gender, age, nationality, handedness) and included three experiments presented in the following order: (1) an Implicit Association Task, (2) a FBR, and (3) a HLT. More details about the procedures and the specific tasks are provided in Supplementary Material.

#### Implicit Association Task

According to Greenwald et al. (1998), participants classified stimuli corresponding to two ethnic groups (in-group: light-skinned faces; out-group: dark-skinned faces) and attributes of positive and negative values (pleasant and unpleasant words) through two key presses. The experiment consisted of seven blocks. The two test blocks presented 48 trials each. The *d* score, which expresses the strength of the automatic association between pleasant/unpleasant words and in-group/out-group stimuli categories, was calculated with an improved scoring algorithm (Greenwald et al., 2003).

#### Full Body Rotation Task and Hand Laterality Task

**Figure 1:**
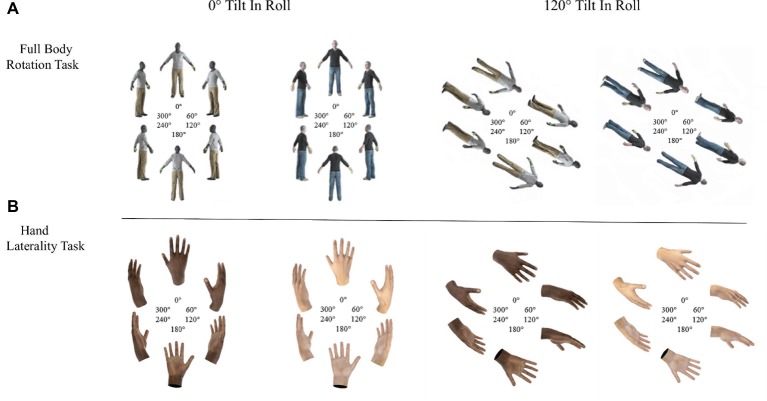
Light- and dark-skinned avatars shown in the Full Body Rotation task (panel **A**) and light- and dark-skinned hands shown in the Hand Laterality Task (panel **B**).

In the FBR, a green sphere was positioned on the left or right hand of 3D models of light- and dark-skinned avatars. Participants responded in a time-sensitive forced-choice task whether the left or right hand was marked (see Figure 1A).

In the HLT, light- and dark-skinned hands were presented and participants responded in a time-sensitive forced-choice task whether a left or right hand was shown (see Figure 1B).

Avatars and hands were rotated along the yaw axis and six different rotation angles, 0, 60, 120, 180, 240, and 180° starting from hands/body at 0 or 120° tilt in roll axis.

For each task, participants underwent a training of 10 trials where a feedback was displayed. Then, a total of 144 stimuli for both the FBR and the HLT were presented (72 depicting light-skinned avatars/hands vs. 72 dark-skinned avatars/hands; 12 different angles of rotation). A fixation cross was shown at the beginning of each trial for 1 s.

#### Data Analysis

RTs associated with the correct responses were the outcome measures. As suggested in Leys et al. (2013), outliers were defined as those >2 absolute deviations around the median and were additionally detected with stem-and-leaf and boxplot displays. Linear mixed models were fitted after checking the assumptions of independence of the residuals and their normal distributions through QQ-Plots and the Shapiro-Wilk Test (*p* > 0.05). Application of a linear mixed procedure was appropriate given the within-person dependence of the data and its longitudinal structure (Fox, 2002). Multilevel modeling also allowed us to take into account each data point (>4,500 observations across all the participants for each task) while adjusting for within-subject and within-group dependence.

### Hypothesis Testing and Results

An implicit racial bias across all participants was observed (D600 score median: 0.55, SD: 0.37). A linear mixed model procedure (Field, 2009; Bates et al., 2014) examined whether the HLT and FBR were suitable for hypothesis testing. Details of these sensitivity analyses are provided in Supplementary Material. For the hypothesis testing, linear mixed models were fit in a stepwise manner according to our hypothesis, and the model’s fit was evaluated by examining the change in −2 log-likelihood, as described in Bliese and Ployhart (2002). Two separate procedures, one for the FBR and one for the HLT, examined whether the implicit bias, indexed by the individual’s mean-centered D600 scores, the skin color (light- and dark-skinned), and the interaction between these two factors modulated RTs. The two final models are reported using the following formula:

$$\begin{array}{l} {\mathrm{RTs}}_{\;\left(\mathrm{FBR, HLT}\right)}=intercept+p+\beta_{1}\left(Implicit\;Bias\right)\\ \;\;\;\;\;\;\;\;\;\;\;\;\;\;\;\;\;\;\;\;\;\;\;\;\;\;\;\;\;\;\;\;\;\;\;\;\;\;+\beta_{2}\left(Skin\;Color\right)\\ \;\;\;\;\;\;\;\;\;\;\;\;\;\;\;\;\;\;\;\;\;\;\;\;\;\;\;\;\;\;\;\;\;\;\;\;\;\;+\beta_{3}\left(Implicit\;Bias\times Skin\;Color\right)+e \end{array}$$

where “*β_x_*” represents the estimated parameters, “*e”* represents the normally distributed residuals, and “*p*” represents the random effects. A random intercept for each participant was modeled for both the HLT (ICC(1) = 0.41, *F*(46, 4,417) = 80.01, *p* < 0.0001) and the FBR (ICC(1) = 0.50, *F*(46, 5,111) = 124.9, *p* < 0.0001). A random slope for Implicit Bias was initially modeled as indicated in Field (2009) but then omitted as it did not improve the model fit when evaluating the change in −2 log-likelihood in the FBR (*χ*^2^(2) = 0.87, *p* = 0.65) and in the HLT (*χ*^2^(2) = 0.22, *p* = 0.90). For the FBR, Implicit Bias (*p* = 0.40), skin color (*p* = 0.81), and the interaction between Implicit Bias and Skin Color (*p* = 0.15) did not modulate participants’ performances. Similarly, in the HLT, RTs were not predicted by the factors Implicit Bias (*p* = 0.35) or Skin Color (*p* = 0.56), nor by the interaction between Implicit Bias and Skin Color (*p* = 0.48). See Supplementary Table S1 for all observed fixed effects. Mean and SD of RTs to dark/light-skinned hands and avatars are reported in Table 1. A separate linear mixed model procedure revealed no modulation of the participants’ nationality on RTs for either task. Details and additional analyses are provided in Supplementary Material.

**Table 1:** RTs in *M* and SD for the HLT and FBR for dark- and light-skinned stimuli, averaged over all angles.

**Hand laterality task**	
Dark-skinned hands RTs: *M* ± SD	952 ± 376
Light-skinned hands RTs: *M* ± SD	943 ± 376
**Full body rotation task**	
Dark-skinned bodies RTs: *M* ± SD	1,514 ± 677
Light-skinned bodies RTs: *M* ± SD	1,499 ± 675

## Discussion

In the present study, we examined how skin color and dispositional implicit negative biases toward people with their own or a different skin color might influence embodied visuospatial egocentric mental transformations. Such perspective taking is presumably at the core of empathic responses (Mohr et al., 2010), which are known to be diminished for out-group members (e.g., Chiao and Mathur, 2010). Contrary to our hypotheses, however, we did not find an influence of the shown skin color nor of individual dispositional implicit biases on the RT in this task.

These findings are not easily commensurable with previous studies. It has been shown that visual capture of the race by means of skin color and implicit biases affect sensorimotor resonance in the context of role playing and perspective taking (Todd et al., 2011, 2012; Peck et al., 2013), action imitation, motor intention encoding, voluntary mimicry (Inzlicht et al., 2012), observation of neutral action (Désy and Théoret, 2007; Molnar-Szakacs et al., 2007; Gutsell and Inzlicht, 2010), as well as sensorimotor and affective mapping of the effects of painful stimulation observed on others as mediated by empathy (Avenanti et al., 2010; Azevedo et al., 2012; Berlingeri et al., 2016). From a neurofunctional point of view, given the well-documented overlap of the neural networks involved in empathy and visual-spatial egocentric perspective taking (Vogeley and Fink, 2003; Santiesteban et al., 2015), we expected these processes to mutually interact during the laterality decisions in the FBR, which require an explicit change of perspective toward the depicted person.

This rather surprising lack of an effect might be in line with a recent study (Désy and Lepage, 2013), which observed no effect of skin color on pure motor resonance as indexed by imitation speed and mu suppression. However, there are important differences to previous studies reporting an effect of skin color which should be considered. These studies typically show another person’s body from a third-person perspective without requiring an *explicit* perspective transformation, thus allowing for a clear self-other distinction in terms of perspective. This might also facilitate the perception of the other person as a whole, including the classification into an in- or out-group member. In fact, in previous studies that investigated sensorimotor processes when the other person’s body parts were seen from a first-person perspective (Farmer et al., 2012; Bufalari et al., 2014), no differences between dark- and light-skinned stimuli were found, at least not on an implicit level (but see Lira et al., 2017 for an exception). At the moment, these interpretations remain largely speculation. Yet, our stimuli were carefully conceived to not be confounded by any emotional or cultural components.

As expected, we also did not find an effect of skin color on the HLT. Again, this is in line with previous studies using multisensory stimulation paradigms from a first-person perspective to induce self-other merging with a light-skinned versus dark-skinned body part (Farmer et al., 2012).

Theoretically, the question remains whether there was truly no effect of skin color, or whether our task and/or our measures were not sensitive enough to uncover subtle differences. For example, a bigger influence could be expected if we presented a real other person rather than an avatar, or a life-sized avatar rather than a small stimulus on a screen. Furthermore, in the case of the rubber hand illusion, no influence of skin color was found for proprioceptive drift (Farmer et al., 2012); yet with a presumably more sensitive measure (time-till-illusion, see Lira et al., 2017), a difference was evidenced. A further critique could be that the study was designed as an online study. Yet, the fact that participants show the classical mental rotation pattern in RTs (i.e., longer RTs for bigger angles) suggests participants solved the task properly, i.e., in a way known from individual lab testing. Finally, while the sample size seems rather small for an online study, both sensitivity and hypothesis testing analyses were adopted to a multilevel approach which allowed to model more than 4,500 observations for each task and therefore to make robust inferences.

To conclude, we did not find any behavioral evidence for a skin color-dependent difference in two classical mental imagery tasks. Yet, it would still be worth to replicate this study in a controlled laboratory setting in order to exclude the variability in the RTs that could be explained by the use of participants’ different hardware. Moreover, future studies should aim at looking at neurofunctional correlates of laterality judgments to dark-skinned *versus* light-skinned bodies, to uncover a potentially differential recruitment of distinct neural sources.

## Ethics Statement

As approved by Kantonale Ethikkommission, Kanton Zürich (BASEC Nr: Req-2018-00727) in the Clarification of responsibility, this study does not fall within the scope of the Human Research Act (HRA). The study was carried out with written informed consent from all the subject. The informed consent was displayed before to start the online survey. All subjects gave written informed consent in accordance with the Declaration of Helsinki.

## Author Contributions

GS and BL designed the experiment. GS and HS programmed the experiment. HS created the stimuli and collected the data. GS and BL collected and analyzed the data. BL, GS, and PB interpreted the results and drafted the manuscript. All the authors proof read and approved the final version of the manuscript.

### Conflict of Interest Statement

The authors declare that the research was conducted in the absence of any commercial or financial relationships that could be construed as a potential conflict of interest.

## Footnotes

^1^https://www.psytoolkit.org
