# Supplementary material for: Putting Yourself in the Skin of In- or Out-Group Members: No Effect of Implicit Biases on Egocentric Mental Transformation
Source: Front Psychol. 2019 Jun 14;10:1338. doi: 10.3389/fpsyg.2019.01338 (PMC6606962; doi:10.3389/fpsyg.2019.01338)
Supplement: Supplementary file 1 [file Data_Sheet_1.PDF]

Supplemental material

Methods.

Procedure. The study was configured as an online study. Participants were provided with a link to the online survey, which they could access using a personal computer or a laptop. Two versions of the survey were created: one in German, the other in Italian. Participants were native speakers of Italian or German and were provided with the corresponding versions. Instructions and materials were delivered according to their mother tongue. Only light-skinned and Caucasian participants, reporting no African nor Asian ancestries, participated to the study after signing the online informed consent form. They were 47 right-handed individuals (Male: 24, Female: 23; *M* = 30.83, *SD* = 10.26, Italian speakers: 24, German speakers: 23).

IAT. The IAT consisted of 20 pictures of dark-skinned and light-skinned people paired with words associated with positive (e.g. love, peaceful) or negative (e.g. war, horrible) concepts. Stimuli can be visualised and downloaded at the following website: https://osf.io/JRvg8/. These stimuli were selected as they are the standard pictures used in the IAT for assessing implicit racial bias. Participants' implicit racial bias was measured by the response time and accuracy of categorizing the pictures with a positive concept relative to a negative concept.

The IAT consisted of seven blocks according to Greenwald et al., 2003. The stimulus remained on the screen until a response was given. Each block started with detailed instructions. Participants were instructed to respond as quickly and accurately as possible with the keys ‘f’ and ‘j’ on the keyboard.

In the first two blocks, pictures and words were presented separately 20 times each and had to be classified as either positive/negative (words) or dark-skinned and light-skinned with the keys ‘f’ for positive and ‘j’ for negative concepts in block one and with the keys ‘f’ for light-skinned (white) and ‘j’ for dark-skinned (black) bodies in block 2.

In blocks three and four, the concepts of white and positive words, respectively, were presented on the left (key ‘f’ had to be pressed) while black and negative words were shown on the right (key ‘j’ had to be pressed). Blocks three and four consisted of 80 trials in total. In these blocks, the association strength between the concepts ‘good’ and ‘white/’bad’ and ‘black’ were measured. In block five, pictures had to be categorized, but with the response to white (right, key ‘j’) and black (left, key ‘f’) switched. In blocks six and seven, black or positive words were presented on the left side (key ‘f’), while white or negative words were presented on the right (key ‘i’). Those last two blocks consisted of 80 trials in total as well. In these blocks, the association strength between the concepts ‘bad’ and ‘white’/’good’ and ‘black’ were measured. Following an incorrect response, a red ‘X’ popped up immediately. An incorrect response was defined as the misclassification of a picture or a word. Responses longer than 10 s were excluded and latencies of incorrect responses were replaced with the block mean and added a penalty

of 600 ms, according to the improved algorithm to calculate the IAT value proposed by Greenwald et al. (2003). The IAT value was calculated with the improved scoring algorithm: The mean difference in RTs between the incongruent and congruent blocks was divided by the pooled standard deviation (*SD)* of all correct trials so that positive IAT scores indicate an implicit preference for light-skinned persons and negative IAT scores indicate an implicit preference for dark-skinned persons.

Full Body Rotation task. Participants were shown either dark or light-skinned avatars displaying a sphere on either the right or left hand. They were instructed to judge as accurately and quickly as possible whether the sphere was positioned on the right hand, by pressing the right key with the right hand (“f” key), or on the left one, by pressing the left key with the left hand (“j” key). Stimuli were presented until a response was given or for a maximum of 5 seconds.

Hand Laterality Task. Left and right hands were presented at the centre of the screen. Participants were required to indicate as accurately and quickly as possible whether a right hand (with the right hand “f” key) or a left hand (with the left hand, “j” key) was shown. As in the FBR, stimuli were presented until a response was given or for a maximum of 5 seconds.

Notably, the same key presses for the three tasks were used. These are “f” for the answers provided with the left hand and “j” for those provided with the right hand. These two keys were chosen due to their equidistance from the two keys located in the middle of the keyboard “g” and “h”.

Sensitivity analysis. A well-established linear mixed model procedure (Bates et al., 2014; Field, 2009; Fox, 2002) examined whether the HLT and FBR were suitable for hypothesis testing. We inspected whether we could: a) find specific differences in the RTs for the two tasks, which indicate that the underlying cognitive processes dissociate; b) replicate and add robustness to the well-known effect of amplitude of the angle of rotation (small vs large) and c) identify whether this effect of angle of rotation was stronger for one of the two tasks. Therefore, we considered the following three factors in the final model: “Task” (HLT vs FBR), “Angle of rotation” (Small vs Large) and the interaction “Task by Angle of Rotation”. This procedure confirmed a strong dissociation between the performances on the two tasks, whereby slower RTs were observed for the HLT in comparison with the FBR (effect of Task: $b = 762.70$, 95% CI: 737.83 to 787.56, $t(46, 9400) = 60.11$, $p < 0.0001$). A well-known effect of angle of rotation was replicated consistently for the two tasks ($b = 60.72$, 95% CI: 37.52 to 83.92, $t(46, 9396) = 5.13$, $p < 0.0001$). In particular, larger compared to smaller angles of rotation were accompanied by slower RTs. However, this discrepancy was found to be greater for the HLT rather than FBR, as

confirmed by a significant interaction of Task by Angle of Rotation (*b* = -18.91, 95% CI: -363.90 to -295.56, *t*(46, 9396) = -18.91, p < 0.0001).

Table 1. ***Summary of the results (fixed effects) for the Hand Laterality Task and Full Body Rotation Task analysis***

Hand Laterality Task - Fixed Effects	*b*	*SE*	*df*	*t*	*p*
(Intercept)	1474.66	79.86	46	18.466	<0.001
Implict Racial Bias	255.65	272.64	46	0.938	0.354
Skin Color	-10.25	17.78	4233	-0.576	0.564
Implicit Racial Bias * Skin Color	-44.9	60.32	4233	-0.744	0.457
Full Body Rotation Task - Fixed Effects	*b*	*SE*	*df*	*t*	*p*
(Intercept)	966.892	45.055	46	21.46	< 0.001
Implict Racial Bias	-132.998	157.948	46	-0.842	0.405
Skin Color	-1.949	8.081	5106	-0.241	0.809
Implicit Racial Bias * Skin Color	-40.482	28.309	5106	-1.43	0.153

Data Analysis & Results.

The effect of nationality. A linear mixed model procedure inspected whether the nationality of the participant was affecting RTs in the FBR and HLT. The final models are reported using the following formula:

RTs (FBR, HLT) = *intercept* + *p* + β_1 *(Nationality)* + *e*.

where "β_x" represents the estimated parameters, "*e*" represents the normally distributed residuals, and "*p*" represents the random effects. No significant effects were found for the FBR (p = 0.28, Italians RTs: *M* = 989 msec, *SD* = 406 ; Germans RTs: *M* = 893 msec, *SD* = 315) and for the HLT (p = 0.17, Italians RTs: *M* = 1586 msec, *SD* = 690; Germans RTs: *M* = 1404 msec, *SD* = 642)

An additional linear mixed procedure further considered the factors skin color and implicit racial bias, and the interactions between these three factors. No significant effects were observed and the fixed effects are reported in table 3

Table 3. ***Summary of the results (fixed effects) for the Hand Laterality Task and Full Body Rotation Task analysis considering the factor Nationality and its interactions with the other factors.***

Hand Laterality Task Fixed Effects	***b***	***SE***	***df***	***t***	***p***
(Intercept)	1362.86	109.426	36	12.455	< 0.001
Implict Racial Bias	517.343	426.303	36	1.214	0.233
Skin Color	-4.005	24.373	4336	-0.164	0.869
Nationality	253.455	160.576	36	1.578	0.123
Implicit Racial Bias * Skin Color	-13.666	94.663	4336	-0.144	0.885
Implicit Racial Bias * Nationality	-611.947	564.114	36	-1.085	0.285
Skin Color * Nationality	-10.73	36.151	4336	-0.297	0.767
Implicit Racial Bias * Skin Color * Nationality	-41.278	125.993	4336	-0.328	0.743
Full Body Rotation Task Fixed Effects	***b***	***SE***	***df***	***t***	***p***
(Intercept)	909.463	64.085	39	14.191	<0.001
Implict Racial Bias	-282.139	252.09	39	-1.119	0.27
Skin Color	-1.944	11.513	5158	-0.169	0.866
Nationality	102.684	91.31	39	1.125	0.268
Implicit Racial Bias * Skin Color	17.852	45.597	5158	0.392	0.695
Implicit Racial Bias * Nationality	165.57	329.535	39	0.502	0.618
Skin Color * Nationality	3.997	16.349	5158	0.244	0.807
Implicit Racial Bias * Skin Color * Nationality	-94.488	59.183	5158	-1.597	0.11

Follow up online survey

A follow-up online survey was conducted to answer some concerns raised in the peer review process. Seventeen German speaking, light-skinned and Caucasian participants were recruited, none reporting African or Asian ancestries. The experiment addressed whether the order of the task, political attitude, and color preference would affect RTs in the FBR and HLT.

For all the analyses, RTs associated with the correct responses were the outcome measures. Outliers were defined as those > 2 absolute deviation around the median and were additionally detected with stem-and-leaf and boxplots displays (Leys et al., 2013). Linear mixed models were fitted after checking the assumptions of independence of the residuals and their normal distributions through QQ-Plots and the Shapiro-Wilk Test ($p > 0.05$).

Order of the tasks. In the main online survey, the sequence of the tasks was not counterbalanced. The FBR always preceded the HLT. Here, the order of the administration of the tasks was reversed, so that the HLT preceded the FBR. We implemented two separate mixed model procedures, one for the HLT and FBR, comparable to the main analyses reported in the manuscript (section: Hypothesis testing and results). The final models are reported using the following formula:

$$RTs_{(FBR,\ HLT)} = \text{intercept} + p + \beta_1\ (\text{Skin color}) + e.$$

where “β_1” represents the estimated parameters, “e” represents the normally distributed residuals, and “p” represents the random effects.

2331 observations for the FBR and 2156 for HLT were generated. No significant effects of skin color were found for the FBR (dark-skinned body RTs: M = 821; SD = 310; light-skinned body RTs: M = 810, SD = 315) and HLT performance (dark-skinned hand RTs: M = 1342, SD = 658; light-skinned hand RTs: M = 1368, SD = 676).

Political Attitude. Political attitude was measured by means of a VAS where participants had to move the cursor to the rating point (1 to 9) that they felt most appropriate to describe their political orientation (1 corresponding to extreme left and 9 extreme right). The mean score was 3.2, SD = 1.3, range = 2-6. Linear mixed models were fitted to investigate the relationship between the factor Political Attitude (treated as continuous variable) and Skin Color. The two final models are reported using the following formula:

$$RTs_{(FBR,\ HLT)} = intercept + p + \beta_1\,(Political\ Attitude) + \beta_2\,(Skin\ color) + B_3\,(Political\ Attitude*Skin\ color) + e.$$

where “β_x” represents the estimated parameters, “e” represents the normally distributed residuals, and “p” represents the random effects. A random intercept for each participant was modelled for both FBR (ICC(1) = 0.21, F(16, 2314) = 38.52, p. < 0.0001) and the HLT (ICC(1) = 0.17, F(16,2139) = 26.46 , p < 0.0001). For the HLT, Political Attitudes (p = 0.77), Skin color (p = 0.5), and the interaction Political*Skin color (p = 0.7) do not modulate RTs. Analogously, for the FBR these factors (Political Attitudes, p = 0.6; Skin color, p = 0.35; interaction Political Attitudes*Skin color, p = 0.56) did not modulate RTs. Figure 2 summarizes these results for the FBR.

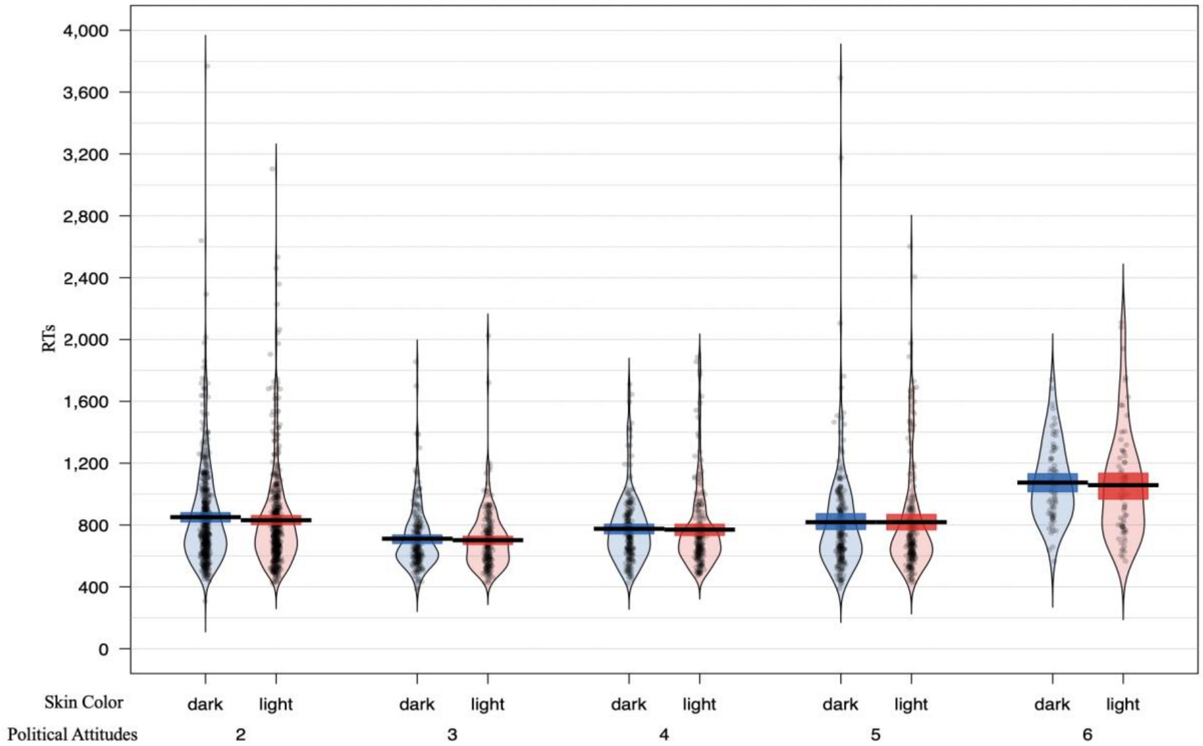

Figure 2. Effects of Political Attitude on RTs in the FBR. Mean RTs and 95% CI are shown for dark and light skinned bodies.

Color preference. In the same online follow-up survey, we inspected whether color preference might affect RTs in the two tasks. Participants were asked to order the colors red, black, blue, white, and green according to their individual preferences. We calculated the difference between the position within the list of the black color and the position within the list of the white color. Individual scores could range from -4 (extreme preference of black color; black color in the first position and the white color in the last position) and +4 (extreme preference of white color; black color in the last position and white color in the first position). The mean score across the participants was -0.70 and *SD* was 2.26. Linear mixed models were fitted and Color Preference has been treated as a continuous variable. Final models are reported using the following formula:

$RTs_{(FBR,\ HLT)}$ = *intercept* + *p* + β_1 *(Color Preference)* + β_2 *(Skin color)* + B_3 *(Political Attitude*Skin color)* + *e*.

where "β_x" represents the estimated parameters, "*e*" represents the normally distributed residuals, and "*p*" represents the random effects.

For the FBR we found no effects of Color Preference (p = 0.692) or of Skin Color (p = 0.59), nor an interaction between Color Preference and Skin Color (p = 0.61). Figure 3 summarizes these results.

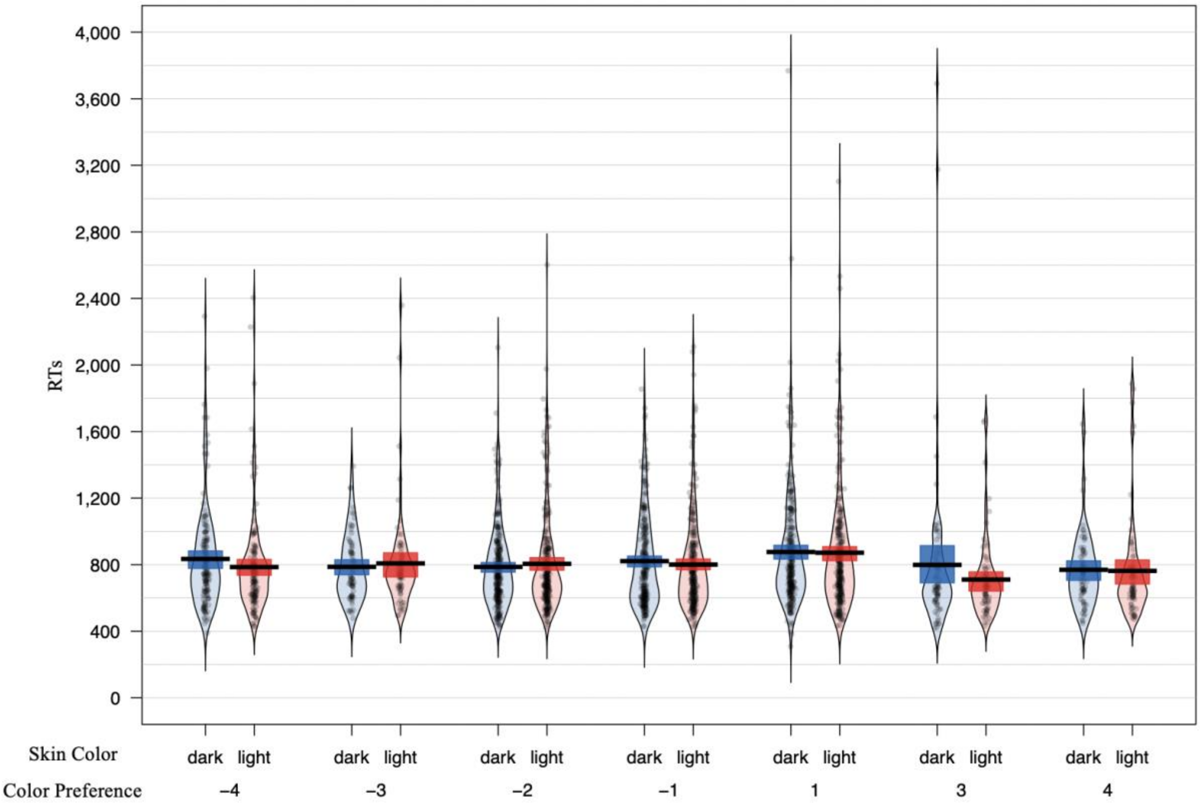

Figure 3. Effects of Color Preference on RTs in FBR. Mean RTs and 95% CI are shown for dark and light skinned bodies.

For the HLT, Color Preference (p = 0.75) , Skin Color (p = 0.13), and the interaction Color Preference * Skin Color (p = 0.07) did not yield significant results.

References

Bates, D., Mächler, M., Bolker, B., & Walker, S. (2014). Fitting Linear Mixed-Effects Models using lme4. *ArXiv:1406.5823 [Stat]*. Retrieved from http://arxiv.org/abs/1406.5823

Field, A. (2009). Discovering statistics using R (3rd ed.). Sage.

Fox, J. (2002). Linear mixed models-appendix to an R and S-PLUS companion to applied regression. *Relation*, *10*, 5261. Retrieved from Scopus.
